# Small Punch Testing of a Ti6Al4V Titanium Alloy and Simulations under Different Stress Triaxialities

**DOI:** 10.3390/ma17174203

**Published:** 2024-08-25

**Authors:** Kun Wang, Xilong Zhao, Zeyu Cao

**Affiliations:** 1 School of Civil Engineering, Lanzhou Jiaotong University, 88 Anning West Road, Anning District, Lanzhou 730070, China; wangkun@mail.lzjtu.cn; 2 School of Materials Science and Engineering, Lanzhou Jiaotong University, 88 Anning West Road, Anning District, Lanzhou 730070, China; czy18803342240@163.com

**Keywords:** Ti6Al4V titanium alloy, mechanical property, stress triaxiality, small punch test

## Abstract

The mechanical properties of local materials subjected to various stress triaxialities were investigated via self-designed small punch tests and corresponding simulations, which were tailored to the geometry and notch forms of the samples. The finite element model was developed on the basis of the actual test method. After verifying the accuracy of the simulation, the stress, strain, and void volume fraction distributions of the Ti6Al4V titanium alloy under different stress states were compared and analyzed. The results indicate that the mechanical properties of the local material significantly differ during downward pressing depending on the geometric shape. A three-dimensional tensile stress state was observed in the center area, where the void volume fraction was greater than the fracture void volume fraction. The fracture morphology of the samples further confirmed the presence of different stress states. Specifically, the fracture morphology of the globular head samples (with or without U-shaped notches) predominantly featured dimples. Modifying the specimen’s geometry effectively increased stress triaxiality, facilitating the determination of the material’s constitutive relationship under varying stress states.

## 1. Introduction

Ti6Al4V titanium alloys are extensively utilized in various industries, such as rail transit, aerospace, and medical equipment, owing to their exceptional qualities, including high specific strength, excellent corrosion resistance, and superior mechanical properties at high temperatures [1,2,3]. This alloy consists of both an α-phase and a β-phase at room temperature, making it a crucial material in engineering applications [4,5]. Its global usage and market share exceed 50%, indicating its widespread adoption worldwide. Considering that titanium alloys and their welded joints are subjected to diverse environmental conditions during service, assessing the localized mechanical properties of these metals and their joints is highly important.

The mechanical properties of a titanium alloy and its joints are influenced by various aspects of the welded joint. Extensive research has been conducted by scholars worldwide, resulting in significant achievements in understanding the local mechanical properties of these materials. However, accurately testing the local mechanical properties requires the precise geometry of the indenter and high accuracy of the load and displacement sensor. In industrial production, the local mechanical properties of materials and their joints are typically characterized via shear punch tests or small punch tests (SPTs) with millimeter-level indenters. Zhao investigated the stress triaxiality and void volume fraction distribution along the path through the thickness direction of TC4 titanium alloy samples. The results revealed significant damage evolution at the notch root area and the area under the indenter edge [6]. Zhao and Wang studied the mechanical properties of laser-welded joints for TC4 titanium alloys via shear punch tests and introduced differences in the microstructure and mechanical properties of each area for welding residual stress simulation [7]. Tan et al. employed the shear punch test to characterize the mechanical properties of narrow gap welded joints of 20Cr2NiMo nuclear rotors. They also established a three-point bending model in commercial finite element software (https://www.ansys.com/) and discussed the J integral value of the welded joint at different distances from the centerline [8]. Kodaira [9] investigated the creep behavior of a polymer block sample under true stress via the SPT method, which effectively characterized the high-temperature mechanical properties of porous polymer materials. Vijayanand [10] investigated the micromechanical properties of a CuCrZr alloy under different heat treatments via the SPT method and used the inverse finite element method to simulate the damage behavior of the alloy via the Gurson–Tvergaard–Needleman (GTN) model. In Shin’s study [11], in situ SPTs were conducted at different temperatures from 0 K to −173 K to investigate the influences of surrounding conditions on the hydrogen embrittlement of various steels. Zhang et al. [12] employed the SPT to investigate the ductile to brittle transition temperature (DBTT) of a tungsten alloy, highlighting the importance of temperature- and orientation-dependent slip behavior on the DBTT. In Wang’s work [13], a combination of finite element simulations and SPTs was utilized to establish databases for samples with varying thicknesses. This research investigated the mechanical properties and fracture model of a wrought Ti6Al4V titanium alloy by SPT, revealing a transition from ductile to semibrittle behavior under hydrogenation [14].

Additionally, the SPT requires only a small volume and is particularly effective when studying the damage and fracture behavior of materials in situations where regular mechanical property testing may be inappropriate [15]. Fan investigated the plastic and damage behaviors of wrought 316LN stainless steel at elevated temperatures via the SPT method to explore the influence of aging on the experimental temperature plastic deformation and damage behavior [16]. Leclerc’s research employs the SPT method and simulation to demonstrate the reliability of acquiring unidirectional stress vs. strain curves for various materials [17]. An integrated experimental system combining SPT and digital image correlation was utilized to determine the mechanical properties of 316 L stainless steel samples [11]. Álvarez [18] investigated SPTs with U-shaped notch samples of various materials by measuring the notch open displacement. Discontinuous methods are employed to investigate the emergence of preferential macrocracks in stress concentration areas. Moreover, the impact of the U-notched depth on the SPT results is evaluated through experiments combined with inverse finite element simulation, which reveals that the DBTT of the 3Cr1MoV alloy is strongly affected by the notched depth-to-thickness ratio [19]. Other studies have investigated the effects of hydrogen on the local mechanical properties and hydrogen embrittlement of various steels via SPT technology with U-notched samples [20]. A350 steel SPT samples with U-shaped and unnotched geometries were examined for the DBTT of the steel, revealing no significant difference in the upper platform and DBTTs on the basis of the sample’s geometrical shape [21]. Pre-notched SPT samples have also been investigated, along with simulation discussions, to determine the dynamic initial fracture toughness of 20MnMoNi55 and T91 to improve the material utilization rates [22]. Furthermore, scientific simulations have been conducted to explore the effects of micromechanical properties via the SPT method [23].

In this work, a self-designed SPT device is utilized to determine the local mechanical properties of the Ti6Al4V titanium alloy. The mechanical properties of the Ti6Al4V alloy under various stress triaxialities are determined by adjusting various parameters, such as the sample geometry. Additionally, inverse finite element technology is employed to validate the accuracy of the model. During the investigation, the evolution of the equivalent stress and the void volume fraction along the path are analyzed and discussed.

## 2. Experiment and Materials

### 2.1. Experiment

**Figure 1:**
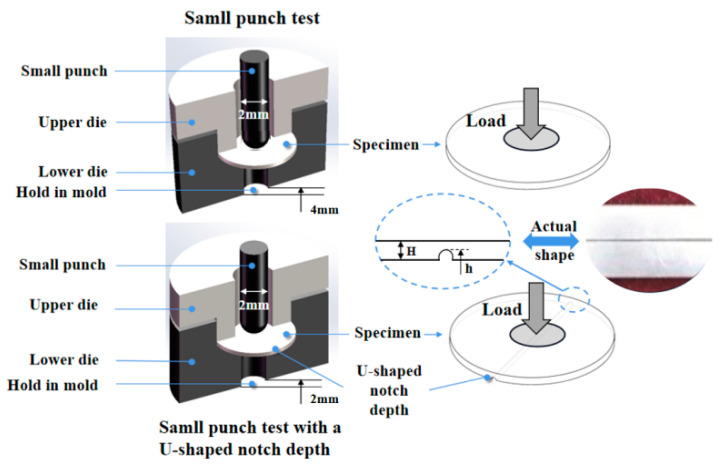
Diagram of the specimen for the small punch test and small punch test with a U-shaped notch.

The experiment utilizes a Ti6Al4V titanium alloy, and its chemical composition is presented in Table 1. Before the start of the SPT experiment, both sides of the sample need to be cleaned and polished. The samples are treated with Kell reagent (4% HF, 10% HNO_3_, and 86% water) to ensure corrosion resistance, and its microstructure is observed via an optical microscope (TXL-3500). The size of the Ti6Al4V titanium alloy specimen is 50 mm × 15 mm × 0.36 mm, and the specimen is free of a U-shaped notch. A U-shaped notch with a depth of 0.24 mm is then created on the back of the sample via a slow wire cutter (XQL-2137). Afterward, both sides of the Ti6Al4V titanium alloy with a U-shaped notch are sanded with sandpaper as a specimen size of 50 mm × 15 mm × 0.6 mm. The SPTs are subsequently conducted on the two samples, as depicted in Figure 1.

**Figure 2:**
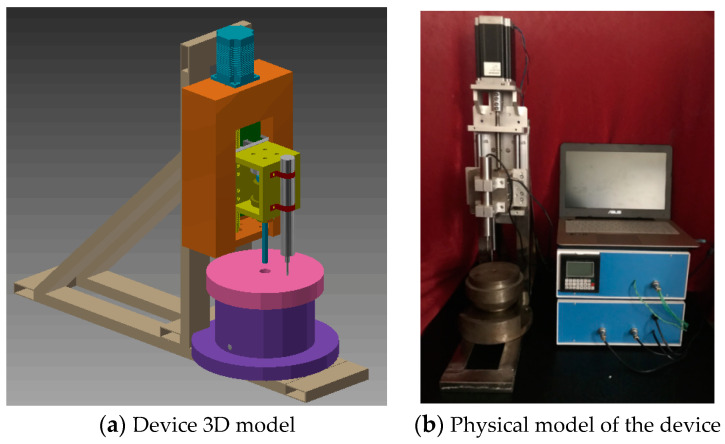
Three-dimensional model and profile display of small punch testing device.

To obtain the mechanical properties of each area within the entire welded joint, a self-designed semiautomatic SPT device is employed. The 3D model and the actual device are presented in Figure 2a,b, respectively. The device comprises a stepper motor drive module, a displacement‒load test module, a sample fixing module, and a semiautomatic control module. First, the specimen fixing module is securely attached to the base of the equipment via bolts. During the fixing and pushing phases, the U-shaped notch area is carefully aligned with the centerline of the spherical indenter, as this alignment significantly affects the accuracy of the experiment and its calculated results. Therefore, precise placement on the sample fixing module is crucial for centering the spherical indenter. The motor drive module is then employed to lower the indenter, whereas the semiautomatic control module regulates the descent speed. The load displacement test module within the device is responsible for collecting load and displacement data, which are recorded by the USB DAQ380i Manual-v3 acquisition card.

### 2.2. Finite Element Model

**Figure 3:**
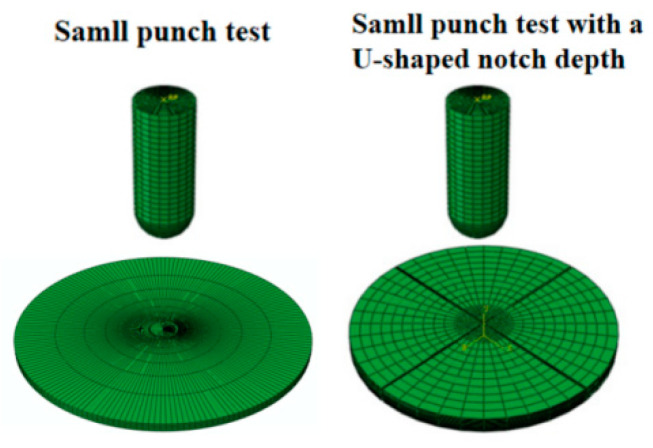
Finite element model of small punch test specimen and small punch test specimen with a U-shaped notch.

For SPTs with or without U-shaped notched samples, a finite element model is established via commercial software. The force-bearing plane of the sample is taken as the XZ direction, with the Y direction serving as the moving direction of the indenter. A friction coefficient of 0.3 is defined between the sample surface and the indenter outside surface, and contact is also defined, as indicated in other works [24]. The element type used is a C3D8R hexahedral mesh, which is discretized into 60,741 elements (with a U-shaped notch) and 50,768 elements (without a U-shaped notch). The pressing speed of the indenter is 0.036 mm/min. Constraint conditions are applied to impose displacement constraints in the X and Y directions, beyond a diameter of 2 mm (with a U-shaped notch) and 4 mm (without a U-shaped notch) at the bottom surface. Thus, both SPTs with and without a U-shaped notch are static load tests, and the GTN damage model is used to describe the plastic damage behavior of the Ti6Al4V titanium alloy. The stress distribution in the center area of the sample and its damage evolution are compared. The finite element models of the two different experimental methods are shown in Figure 3.

### 2.3. GTN Damage Model

The damage model used in the calculation of local mechanical properties is the modified GTN damage model, where the surface potential function of a spherical void shape is described by Gurson [25,26]. Tvergaard [27,28] has made continuous improvements to this model, as shown in Equation (1):

(1) $$\Phi (\sigma ,\sigma_{M},f)={\left(\frac{\sigma_{\mathrm{eq}}}{\sigma_{y}}\right)}^{2}+2fq_{1}\cosh\left(\frac{3q_{2}\sigma_{H}}{2\sigma_{y}}\right)-(1+q_{3}f^{2})$$

*σ*_eq_ is the equivalent stress, *σ*_y_ is the yield stress, and *σ*_H_ is the hydrostatic pressure. *q*_1_, *q*_2_, and *q*_3_ are parameters introduced by Tvergaard. The calculation formula of the void volume fraction is described as

(2) $$f=\{\begin{array}{cc} f & f\leq f_{c}\\ f_{c}+\frac{1/q_{1}-f_{c}}{f_{f}-f_{c}}(f-f_{c}) & f\geq f_{c} \end{array}$$

In the formula, *f* is the void volume fraction. *f*_c_ is the critical void volume fraction. *f*_f_ is the fracture void volume fraction. The volume fraction variation d*f* of the void is calculated as

(3) $$df=df_{\mathrm{growth}}+df_{\mathrm{nucleation}}$$

In the formula, d*f*_growth_ is the volume fraction change in the void when the void grows up, and d*f*_nucleation_ is the volume fraction change in the void when the void is nucleated. d*f* is the variation in the total void volume fraction. The calculation formula of the stress triaxiality is

(4) $$T=\frac{\sigma_{H}}{\sigma_{\mathrm{eq}}}$$

It is crucial to determine the parameters of the GTN damage model to obtain accurate results. The initial void volume fraction (*f*_0_) is defined as the void volume fraction existing in the specimen before the local mechanical property test. Firstly, the *f*_0_ of the specimen is observed via a SZ745 bulk optical microscope. The initial void volume fraction *f*_0_ in the sample is quantified via WPS 2020 software. Metallographic photos of the samples are processed with black-and-white gray processing before statistical analysis, and the void volume fraction in the base metal is then determined. Multiple metallographic images are obtained, and the average value is calculated. Statistical analysis reveals that the initial void volume fraction *f*_0_ of the Ti6Al4V titanium alloy sample used in this study is 0.00058 [14].

**Figure 4:**
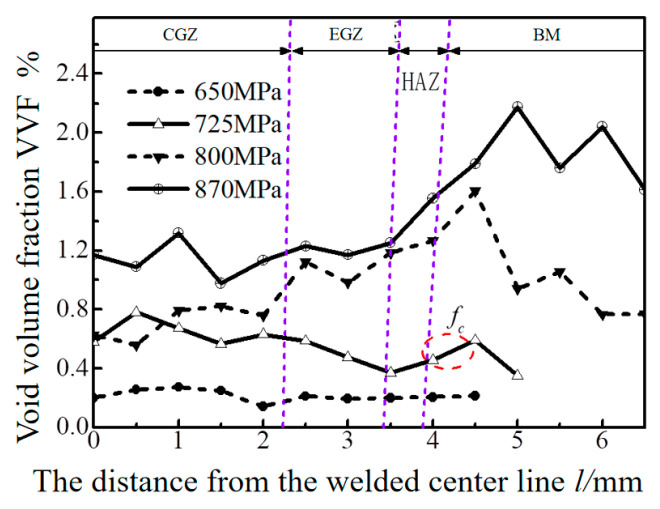
Damage evolution of laser welded joints of Ti6Al4V titanium alloy [14].

The critical void volume fraction (*f*_c_) refers to the void volume fraction in the local mechanical property sample gradually increasing to the critical value as the stress during the SPT test increases. Alternatively, the void volume fraction in a sample increases rapidly when it exceeds the critical void volume fraction (*f*_c_). To investigate the difference in damage behavior among different areas of the entire welded joint, Zhang et al. conducts a study on the damage behavior (void volume fraction) of the tensile samples of the Ti6Al4V titanium alloy laser-welded joints under different loads, as shown in Figure 4. The critical void volume fraction (*f*_c_) in the base metal is represented by a red wire frame in Figure 4, and its value is determined to be 0.005.

The parameters of the GTN damage model are as follows: *q*_1_ = 1.5, *q*_2_ = 1, *q*_3_ = 2.25 [29], the work hardening exponent (*n*) is 0.13, the nucleation equivalent plastic strain (*ε*_n_) is 0.3 [30], and the standard deviation (*S*_n_) is 0.1 [31].

## 3. Results and Discussion

### 3.1. Microstructure and Mechanical Properties

**Figure 5:**
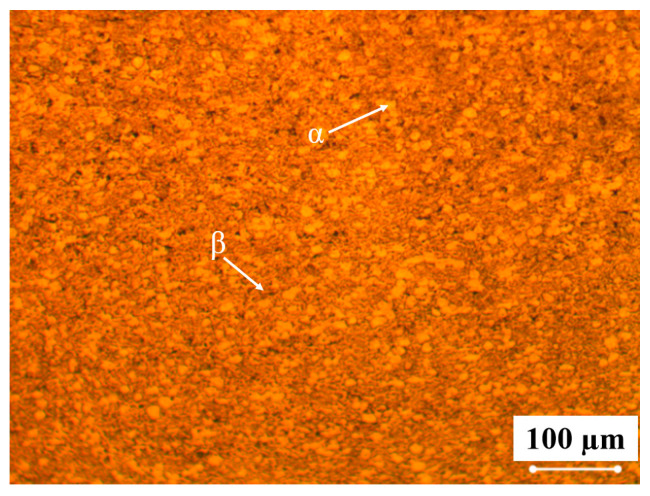
Microstructure of Ti6Al4V titanium alloy.

Prior to the local mechanical property test, the microstructure of the Ti6Al4V titanium alloy consisted of an α phase and a β phase, as shown in Figure 5. The microstructure reveals relatively small and dispersed grain sizes ranging from 5 µm to 25 µm.

**Figure 6:**
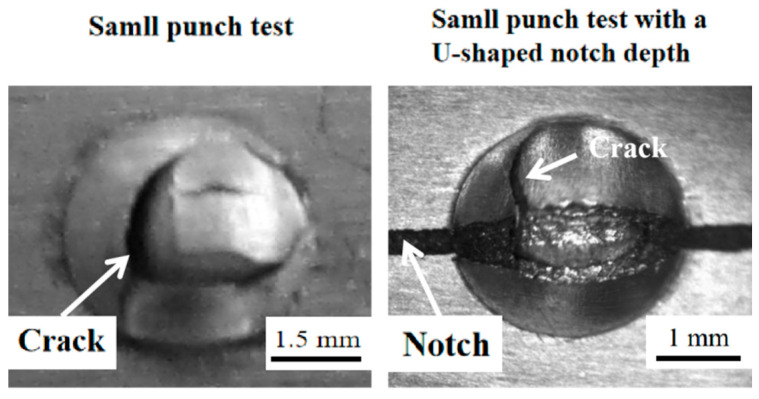
Macroscopic fracture of small punch test and small punch test specimens with a U-shaped notch.

The macroscopic fracture morphology of the Ti6Al4V titanium alloy in the SPT samples, with or without a U-shaped notch, is shown in Figure 6. There are significant differences in the macroscopic fracture morphology between the SPT sample and the SPT sample with a U-shaped notch. This can be attributed to the fact that the stress states in these two different tests do not appear to be quite different. The fracture morphology of the SPT sample exhibited an uncertain fracture path and a coarse surface, indicating typical ductile fracture characteristics, as depicted in Figure 6. Additionally, it is clear that the SPT sample mainly experiences localized fractures in the area under the edge of the indenter, with numerous macroscopic cracks present in this region. Meanwhile, the fracture morphology of the small punch testing specimen with a U-shaped notch shows that the fracture path and surface are certain, typical ductile fracture characteristics, as shown in Figure 6. In this case, the damage evolution of the sample with the U-shaped notch primarily occurs in the notch root area, where numerous macroscopic cracks form.

**Figure 7:**
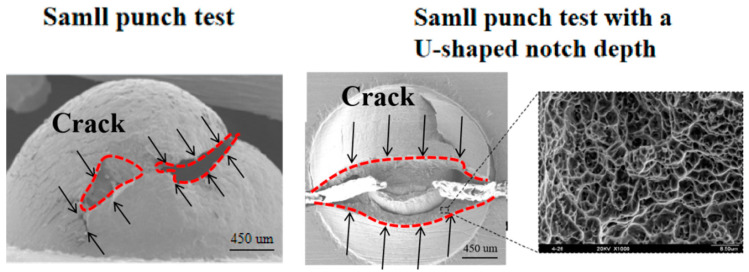
Fracture morphology of small punch test and small punch test with a U-shaped notch.

Figure 7 shows that the degree of alignment of the U-shaped notch has a substantial influence on the test results and the mechanical properties of the sample during fracture. The stress state of the material differs between the two methods employed for local mechanical property testing. Further investigation reveals that the fracture morphology of both SPT samples, with or without a U-shaped notch, consists mainly of dimples, indicating a three-dimensional tensile stress state. However, the fracture morphology of the U-shaped notch sample indicates that the stress state in the notch tip area is primarily normal stress. Therefore, the fracture morphology of these two samples confirms that significant differences in the stress state occur in the thickness direction.

### 3.2. Finite Element Calculation

**Figure 8:**
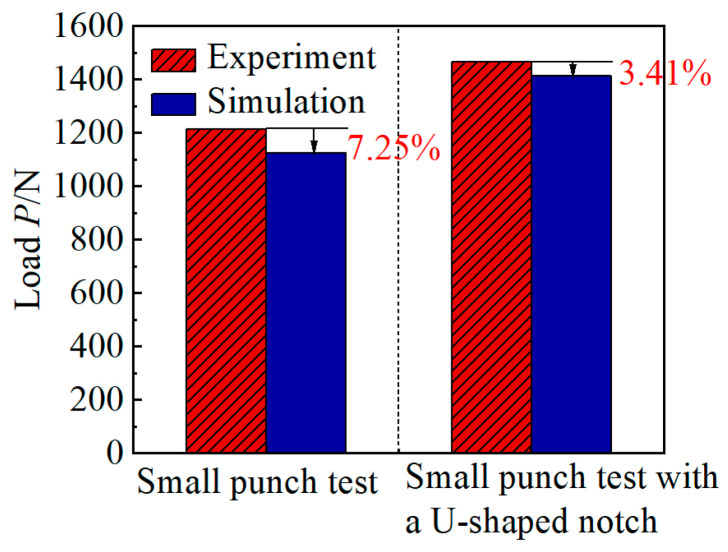
Comparison of margin of error between small punch test specimen and the small punch test specimen with U-shaped notch obtained by experiment and simulation.

The comparison between the max load from two local mechanical property methods and simulation is shown in Figure 8. It can be seen from Figure 8 that the maximum error accuracy of the maximal load obtained by the experiment and simulation is 7.25% and 3.41%, respectively. This phenomenon verifies the accuracy of the finite element model. For the small punch test specimen, the crack propagation at the area below the edge of the pressure head after the peak load reaches 1214 N, and the indenter displacement is 4.78 mm by simulation. For the U-shaped notch specimen, the crack propagation at the notch root area after the peak load reaches 1465 N, which leads to the failure of the specimen. This phenomenon is mainly due to the crack propagation path and the metal resistance to crack propagation in the notch tip area.

**Figure 9:**
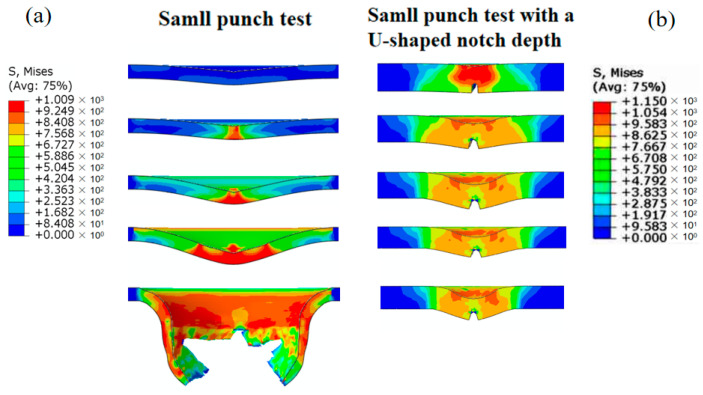
Mises stress distribution at different iteration steps. (**a**) Small punch test without a U-shaped notch. (**b**) Small punch test with a U-shaped notch.

**Figure 10:**
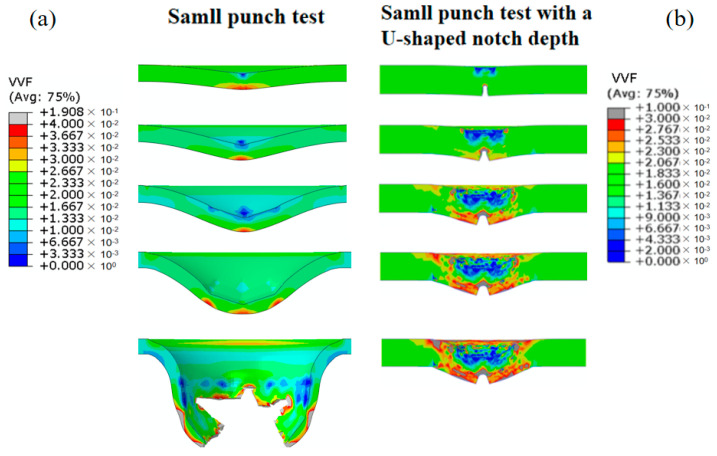
Evolution distribution of Mises stress under different iteration steps. (**a**) Small punch test without a U-shaped notch. (**b**) Small punch test with a U-shaped notch.

On the basis of the verification of the accuracy of the simulation model, the von Mises equivalent stress distribution of the SPT sample is 1009 MPa, as depicted in Figure 9. The value gradually increases with the increasing displacement of the indenter. However, as the displacement reaches its peak value, the von Mises equivalent stress decreases. On the other hand, the von Mises equivalent stress distribution of the SPT sample with a U-shaped notch is more complex, as shown in Figure 10. The stress concentration primarily occurs in the notch root area and below the indenter edge. This indicates that more energy is consumed in the U-shaped notch area of the SPT. Final failure occurs at the notch root area when the stress reaches its peak value, which is consistent with the experimental results. Furthermore, the calculation results are also reflected in the void volume fraction at the notch root area.

To further analyze the stress and damage distribution of the sample in the SPT with a U-shaped notch, Figure 9 reveals a significant decrease in internal damage as the critical void volume fraction is reached with increasing pressure on the indenter. The maximum stress experienced by the material in this region is 1150 MPa. The evolution of the void volume fraction is shown in Figure 10, where it can be observed that the void volume fraction gradually increases with the indenter displacement, particularly in the notch root area. Failure fracture occurs in the localized area of a sample when the critical void volume fraction, *f*_c_, is reached. Moreover, as the indenter continues to lower, a high void volume fraction develops at the notch root and below the indenter edge, exceeding the critical void volume fraction *f*_c_. These findings indicate that different degrees of failure occur preferentially at the notch root area and below the indenter edge in the U-shaped notch sample, which is consistent with other studies [32]. However, the nucleation and growth rates of voids in the Ti6Al4V titanium alloy are slow when the strain is small. Therefore, the damage evolution intensifies significantly when the void volume fraction exceeds *f*_c_. The stress within the titanium alloy in the test area decreases significantly as the void volume fraction increases. The final failure of a sample occurs when the strain corresponding to the void volume fraction in the titanium alloy reaches the fracture strain.

**Figure 11:**
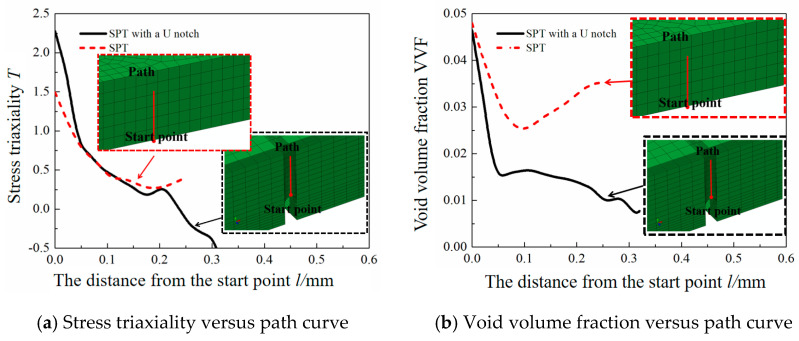
Stress triaxiality and void volume fraction curve of small punch test specimen with or without U-shaped notch.

As depicted in Figure 11a, the SPT with a U-shaped notch reveals a decrease in stress triaxiality in the path direction of the notch root region from 2.28 to −0.64, indicating high three-dimensional tensile stress at the notch root region. Furthermore, at the end of the path, the stress triaxiality value of −0.64 suggests that the metal in the region is under three-dimensional compression. Similarly, in the SPT, the stress triaxiality in the path direction of the notch root region decreases from 1.5 to 0.42. Figure 11b shows that the maximum void volume fraction in the notch root area follows a distribution trend similar to that of the stress triaxiality. Conversely, in the SPT conducts without a U-shaped notch, the void volume fraction at the bottom center surface is highest along the path.

## 4. Conclusions

After establishing the reliability of the self-designed local mechanical device, we conduct SPTs on Ti6Al4V titanium alloy samples both with and without a U-shaped notch while altering the sample geometry. Concurrently, the finite element method is used to simulate the local mechanical property testing procedures. These conclusions have certain reference values for future applications in the industrial field. The following conclusions are drawn:

1. The SPT and its simulation for a Ti6Al4V titanium alloy are executed. The von Mises equivalent stress distributions for the SPT samples with and without a U-shaped notch are determined to be 1009 and 1150 MPa, respectively. For the SPT samples, macrocracks emerge in the U-shaped notch samples as the indenter displacement increases, eventually leading to failure due to the continuous expansion of these cracks. The macroscopic fracture pattern of the sample indicates that material failure is caused primarily by normal stress, confirming the results obtained through flank direction finite element calculations. The combination of the self-designed local mechanical property testing method and inverse finite element simulation offers enhanced characterization of the specimen’s mechanical properties.
2. For the SPT samples, a reduction in stress triaxiality from 1.5 to 0.42 can be observed in the path direction of the notch root region. Moreover, the maximum void volume fraction is situated at the top center surface along the path for these samples. In contrast, the SPT with a U-shaped notch demonstrates a decrease in stress triaxiality from 2.28 to −0.64 in the path direction of the notch root region.

## Figures and Tables

**Table 1:** Chemical composition of Ti6Al4V titanium alloy (wt %).

Al	V	Fe	Si	C	N	H	O	Ti
5.5~6.8	3.5~4.5	≤0.3	≤0.15	≤0.1	≤0.05	≤0.015	≤0.2	Bal.

## Data Availability

The original contributions presented in the study are included in the article, further inquiries can be directed to the corresponding author.
